# Low incidence of COVID-19 in children and adolescent post-liver transplant at a Latin American reference center

**DOI:** 10.6061/clinics/2020/e1986

**Published:** 2020-05-25

**Authors:** Uenis Tannuri, Ana Cristina Aoun Tannuri, Mariana Nutti de Almeida Cordon, Helena Thie Miyatani

**Affiliations:** Divisao de Cirurgia Pediatrica, Unidade Pediatrica de Transplante de Figado e Laboratorio de Pesquisa em Cirurgia Pediatrica (LIM 30), Faculdade de Medicina FMUSP, Universidade de Sao Paulo, Sao Paulo, SP, BR.

Since the report of the first patient in Brazil with Coronavirus disease 2019 (COVID-19), in São Paulo city, on February 26, 2020, there have been concerns about COVID-19 in select patient populations, mainly solid organ transplanted patients, who are normally maintained in an immunosuppressed state to avoid organ rejection. Despite the universal knowledge that children and adolescents with COVID-19 have milder clinical manifestations than other groups of patients (such as those >60 years of age) (1), we have always dedicated great care to the pediatric patients in the early and late postoperative period after a liver transplant. We have further enhanced our perioperative care with special emphasis on diagnosing possible COVID-19 or any associated morbidity. Our concern was based on the rising occurrence of COVID-19 with severe acute respiratory syndrome (SARS) leading to death in some cases in São Paulo, the most populous city of Latin America.

Our hospital is one of the most important reference centers for the treatment of pediatric liver diseases and pediatric transplantation in Latin America, with extensive experience in patients with acute liver failure (2). Our liver transplantation program started in September 1989 (3). The main objective of this presentation is to report our experience of detecting COVID-19 in liver transplant patients in our ambulatory outpatient clinic.

Since the start of our program, 845 children have undergone liver transplant as a treatment for various terminal liver diseases at our institution: 395 were living donor liver transplants and 447 were from cadaveric donors. The main indication for liver transplant were biliary atresia and cirrhosis (387 patients), other forms of cirrhosis including autoimmune diseases (133 patients), fulminant hepatic failure (121 patients), and diverse causes, including diffuse hepatoblastoma needing chemotherapy and metabolic diseases (204 patients). All patients received immunosuppressive drugs that consisted of a calcineurin inhibitor, either cyclosporine, from 1989 to 2000, or tacrolimus, from 2001 to present day. Treatment with methylprednisolone (20 mg/kg) was initiated from the moment of graft reperfusion and then gradually tapered in the postoperative period to a maintenance dosage of 0.5 to 1.0 mg/kg of body weight/day. A third drug, mycophenolate mofetil (MMF), was administered solely in cases of repeated episodes of rejection or refractory rejection. MMF or sirolimus was also used as a single-drug immunosuppressant for patients with deterioration of renal function. Additionally, almost all patients received an anti-hypertensive drug, such as an angiotensin-converting enzyme inhibitor (ACEi).

The patients were routinely examined in the ambulatory clinic or were assessed telephonically. We presently have approximately 190 liver transplant patients who are constantly being followed-up at our ambulatory clinic. Those who turned adults are currently being followed-up at an adult service.

Immediately after the first report of COVID-19, in March and April, our patients were routinely checked for any clinical signs or symptoms suggestive of COVID-19. During this period, six patients presented with symptoms consistent with mild upper airway infection and fever; however, among the tested patients, all but one had a negative RT-PCR test. None of the patients, including the patient who tested positive for SARS-CoV-2, developed clinical pulmonary disease. Also, during this period, 169 non-transplant children in our institution, suspected of having COVID-19, were tested for the virus, and 13 (7.7%) had a positive test. All of them presented with mild clinical manifestations and one of them died secondary to a serious genetic syndrome. Finally, for a better understanding of the major differences between children and adults, we were notified that during the same period, 1,251 adult patients were referred to Hospital das Clinicas da Faculdade de Medicina da Universidade de São Paulo and 208 of them were admitted to the intensive care unit.

The first important consideration about the current report is that in a group of patients who theoretically should be at high risk for developing COVID-19 and SARS, none of them had the disease or suffered from complications. Our patients have many risk factors, including immunosuppression with multiple drugs, autoimmune disease, unknown immunological disturbances as seen in some cases of fulminant hepatic failure, and chemotherapy in patients with hepatoblastoma. In general, patients, need treatment with an ACEi to counteract hypertension caused by immunosuppression treatment with calcineurin inhibitors, a treatment considered to be a risk factor for developing SARS COVID-19 in adult patients (4).

The current report concurs a recent letter by D’Antica from Lombardy (Italy), who reported a similar experience with a group of 700 liver transplant children. D’Antica concluded that “immunosuppressed patients are not at increased risk for severe pulmonary disease when compared to the general population. Children under the age of 12 do not develop coronavirus pneumonia, regardless of their immune status, although they get infected and can, therefore, spread the infection.” Also, a conclusion was mentioned as part of a guideline published on May 1st, 2020, in the UK National Institute for Health and Care Excellence, where stated, “COVID-19 usually causes a mild, self-limiting illness in children and young people, even in those who are immunocompromised” (5). Based on these conclusions from Europe and our initial experience described here, we can affirm that during the present coronavirus outbreak there are no reasons to postpone life-saving treatments such as liver transplantation in children.

Finally, some important points need clarification. We have seen a decrease in the supply of cadaveric donors during this pandemic. Should we, therefore, perform living donor liver transplantations? What if healthy young parents of children with terminal liver diseases are available for liver donation? In extreme situations, such as fulminant hepatic failure in children, should we use organs from SARS-CoV-2 positive donors or should we transplant to SARS-CoV-2 positive recipients? There is evidence that the SARS-CoV-2 virus will not be completely eradicated for a while, and we hope that the scientific community will help us to find ways to make correct decisions in such difficult situations.
